# Sequence-based GWAS in 180,000 German Holstein cattle reveals new candidate variants for milk production traits

**DOI:** 10.1186/s12711-025-00951-9

**Published:** 2025-02-04

**Authors:** Ana-Marija Križanac, Christian Reimer, Johannes Heise, Zengting Liu, Jennie E. Pryce, Jörn Bennewitz, Georg Thaller, Clemens Falker-Gieske, Jens Tetens

**Affiliations:** 1 Department of Animal Sciences, University of Goettingen, Burckhardtweg 2, 37077 Göttingen, Germany; 2 Center for Integrated Breeding Research, Department of Animal Sciences, University of Goettingen, Albrecht-Thaer-Weg 3, 37075 Göttingen, Germany; 3 Institute of Farm Animal Genetics, Friedrich-Loeffler-Institut, 31535 Neustadt, Germany; 4 Vereinigte Informationssysteme Tierhaltung w.V. (VIT), 27283 Verden, Germany; 5 Agriculture Victoria Research, AgriBio, Centre for AgriBioscience, Bundoora, VIC 3083 Australia; 6 School of Applied Systems Biology, La Trobe University, Bundoora, VIC 3083 Australia; 7 Institute of Animal Science, University of Hohenheim, 70599 Stuttgart, Germany; 8 Institute of Animal Breeding and Husbandry, Christian-Albrechts-University, 24118 Kiel, Germany

## Abstract

**Background:**

Milk production traits are complex and influenced by many genetic and environmental factors. Although extensive research has been performed for these traits, with many associations unveiled thus far, due to their crucial economic importance, complex genetic architecture, and the fact that causal variants in cattle are still scarce, there is a need for a better understanding of their genetic background. In this study, we aimed to identify new candidate loci associated with milk production traits in German Holstein cattle, the most important dairy breed in Germany and worldwide. For that purpose, 180,217 cattle were imputed to the sequence level and large-scale genome-wide association study (GWAS) followed by fine-mapping and evolutionary and functional annotation were carried out to identify and prioritize new association signals.

**Results:**

Using the imputed sequence data of a large cattle dataset, we identified 50,876 significant variants, confirming many known and identifying previously unreported candidate variants for milk (MY), fat (FY), and protein yield (PY). Genome-wide significant signals were fine-mapped with the Bayesian approach that determines the credible variant sets and generates the probability of causality for each signal. The variants with the highest probabilities of being causal were further classified using external information about the function and evolution, making the prioritization for subsequent validation experiments easier. The top potential causal variants determined with fine-mapping explained a large percentage of genetic variance compared to random ones; 178 variants explained 11.5%, 104 explained 7.7%, and 68 variants explained 3.9% of the variance for MY, FY, and PY, respectively, demonstrating the potential for causality.

**Conclusions:**

Our findings proved the power of large samples and sequence-based GWAS in detecting new association signals. In order to fully exploit the power of GWAS, one should aim at very large samples combined with whole-genome sequence data. These can also come with both computational and time burdens, as presented in our study. Although milk production traits in cattle are comprehensively investigated, the genetic background of these traits is still not fully understood, with the potential for many new associations to be revealed, as shown. With constantly growing sample sizes, we expect more insights into the genetic architecture of milk production traits in the future.

**Supplementary Information:**

The online version contains supplementary material available at 10.1186/s12711-025-00951-9.

## Background

Intensive selection for milk production traits enhanced with improved nutrition and management, as well as reproductive technologies and accelerated by genomic selection (reviewed by [1]) has strongly increased milk production over the years [2]. The Holstein breed is dominant in milk production worldwide. In Germany, the Holstein population alone comprises 2.4 million cows, with an average milk yield of 10,000 kg per lactation [3]. The breeding goal for German Holstein is balanced and includes many traits that can be grouped into milk production, health, fertility, and longevity [4]. This has not always been the case, and although selection for milk production has been successful in increasing milk yield, it has also been associated with a higher incidence of mastitis, metabolic, and reproductive diseases [5]. The relative weight of milk production in total merit indices is decreasing as new traits are continuously added to the breeding goal. However, because production still makes up a substantial part (e.g., 36% in Germany [3]) and genetic progress must be monitored in order to avoid the risk of a further decline in animal health. More extensive knowledge of the genetic architecture of economic traits is needed, especially given that the majority of these traits are complex traits, influenced by many genes and environmental factors.

So far, genome-wide association studies (GWAS) have been successful in the discovery of quantitative trait loci and candidate genes (reviewed by [6]), however, only a few causal variants for economically important traits in cattle have been confirmed [7, 8]. In order to be able to detect potential underlying causal variants, whole-genome sequence (WGS) data and large samples are needed to ensure sufficient power of GWAS [9, 10]. GWAS in cattle is restricted by long-distance linkage disequilibrium (LD) segments [11], due to a small effective population size (*N*_*e*_) caused by intense selection [12], therefore making it hard to pinpoint the true causal variant which may be hidden among the many variants in LD. Another source of difficulty in revealing the true associations is the highly polygenic genetic architecture of quantitative traits, i.e., large number of variants with small effects affecting the trait [13]. Large samples of sequenced animals, required for powerful GWAS, are generally not available. To overcome this, imputation [14] can be utilized as a method to obtain the sequence-dense data. Imputation methods exploit LD patterns among the individuals in the sample and reference dataset and infer the information about untyped variants based on a smaller number of available genotyped markers [15]. Imputation accuracy depends on various factors such as the size of the reference panel, the relationship between the individuals in the reference and sample dataset, imputation software choice, the number of the variants to be imputed, and minor allele frequency (MAF) of variants [16–19]. In cattle, sequence-level imputation is usually performed in two steps, due to higher accuracy obtained when first imputing from a lower to a higher-density SNP chip, and then to sequence level [18]. With the numbers of cattle genotyped and subsequently imputed constantly growing, there is a need for software that can handle such an amount of information. In human studies, several GWAS software [20–22] have been developed to enable analyzing large samples (e.g., tens to hundreds of thousands of individuals).

To exploit the power of large sample sizes in detecting novel causal loci, we carried out GWAS for three milk production traits using imputed sequence data. After obtaining GWAS summary statistics with a mixed linear model approach (MLMA), meta-analysis was utilized to pool the results of different animal groups. For this purpose, we evaluated different meta-analysis approaches implemented in METAL [23]. In addition, we tested two software that enable the use of large sample sizes in GWAS; fastGWA [20] and SAIGE [21]. Genome-wide significant variants were further fine-mapped to identify potential causal associations, which were eventually annotated and ranked based on their functional and evolutionary significance according to Xiang et al. [24]. Candidate gene research was performed for genes located close to potential causal variants. Finally, the percentage of genetic variance explained by the candidate causal variants was calculated to see which proportion of the variance could be attributed to novel candidate variants.

## Methods

### Dataset

The dataset for imputation consisted of 180,217 German Holstein cows, belonging to a larger dataset, with 45,613 SNP markers. Animals were mainly genotyped with various low-density SNP genotyping arrays (see Additional file 1: Table S1) and then imputed to 50K level according to the national genetic evaluation procedure [25], or genotyped with various 50K SNP chips (see Additional file 1: Table S1). The dataset was collected during the KuhVision project that aimed to genotype and phenotype German Holstein cows to establish a large-scale female reference population for genomic evaluation. The phenotypes for milk (MY), fat (FY), and protein yield (PY) in kg were obtained in the form of deregressed proofs (DRPs) [26], produced using the special single-step SNP BLUP model for deregressing genomic estimated breeding values (GEBV) [27]. Reliabilities of DRPs were similar across the animals and traits, therefore weighting was not used in GWAS.

### Imputation

The genomic coordinates of the input genotypes were lifted from the previous bovine reference genome assembly UMD3.1 [28] to the ARS-UCD1.2 assembly [29] with a custom approach that uses conversion tables. The sample of 180,217 cows consisting of 29 autosomal pairs was imputed to sequence level (78,364,991 variants) in a two-step imputation approach using BEAGLE v. 5.2 [30]. The effective population size parameter was set to 1000. The animals were first imputed to high-density (HD) genotype level using the genotype data of 1278 Holstein cows consisting of 585,517 markers [31]. The HD reference panel was phased using BEAGLE v. 5.1 beforehand [32]. In the next step, data were imputed to the WGS level using the multi-breed reference panel from the 1000 Bulls Genome Project Run9 [33]. The reference panel consisted of 5116 cows and bulls of the species *Bos taurus* (see Additional file 1: Table S2). Both imputation steps were performed chromosome-wise, with the samples divided into random groups of approximately equal size (**≈**5255 individuals), due to high computational requirements. The imputed files were indexed afterwards with IndexFeatureFile, GATK v. 4.2.2.0 [34], merged by the sample groups, and multi-allelic variants (SNPs, insertions, and deletions) were split into bi-allelic sites using BCFtools v. 1.14 [35]. As a quality control, the imputed WGS dataset was filtered using the dosage R-squared parameter, a measure of the estimated squared correlation between estimated and true allele dosage (DR2; [36]). Markers imputed with DR2 < 0.75 were removed with BCFtools, leaving 21,812,477 markers for further analyses. The imputed WGS dataset was annotated with VariantAnnotator from the GATK v. 4.2.2.0 using the Ensembl variation database, release 105 [37] imported from dbSNP [38], to account for SNPs without reference SNP cluster ID (rsID).

### GWAS

#### GCTA and METAL

The sample for GWAS consisted of 180,217 WGS-imputed cows with phenotypic observations for MY, FY, and PY. Due to memory restrictions of the used high-performance computing (HPC) cluster, the samples were divided into 4 groups consisting of **≈**45,000 animals each. GWAS was performed using the GCTA software v. 1.93.2 beta [39] applying a mixed linear model approach for all autosomes. Samples were filtered for MAF lower than 0.01 while running the MLMA, leaving 17,256,703 variants for GWAS. The SNP effects were estimated using the following model:

1 $${\mathbf{y}} = {\mathbf{Xb}} + {\mathbf{Zu}} + {\mathbf{e,}}$$

where $\mathbf{y}$ is a vector of DRPs; $\mathbf{b}$ is the vector of fixed effects of the variant tested for the association with each trait; $\mathbf{X}$ is the incidence matrix of $\mathbf{b}$; $\mathbf{u}$ is the vector of polygenic effects with $\mathbf{u}\sim \text{ N }(0,\mathbf{G}{\sigma }_{\text{u}}^{2})$, where $\mathbf{G}$ is genomic relationship matrix (GRM) calculated using 33,009 variants from 50K SNP chip from all autosomal chromosomes, filtered for MAF lower than 0.01, and ${\sigma }_{\text{u}}^{2}$ is a variance of polygenic effects; $\mathbf{Z}$ is the incidence matrix of $\mathbf{u}$; and $\mathbf{e}$ is the vector of residual effects with $\mathbf{e}\sim \text{ N}(0,\mathbf{I}{\sigma }_{\text{e}}^{2})$, with $\mathbf{I}$ being an identity matrix and ${\sigma }_{\text{e}}^{2}$ residual variance. Bonferroni correction was used to set a genome-wide significance threshold, corresponding to a *p*-value of 0.05/number of markers (2.897 × 10^–9^). The Manhattan plots were created with packages readr [40], ggrepel [41], ggplot2 [42], RColorBrewer [43] and dplyr [44] using RStudio v. 4.2.2 [45].

METAL software [23] for meta-analysis was used to merge the GWAS summary statistics of each of the four animal groups per trait. METAL implements two methods, sample size and an inverse-variance-based approach [23]. For simplification, we will refer to the sample size-based approach as the z-score approach throughout the text. We applied both approaches, examining at the same time the impact of additional settings, namely fixed effects or random effects, with and without sample size weighting. Subsequently, the genomic correction was carried out on meta-analyzed files by loading meta-analysis results into METAL, to correct for inflation. Lambda (λ) values were calculated as the median of observed χ^2^ test statistics divided by the expected median of χ^2^ distribution with one degree of freedom.

#### fastGWA and SAIGE

To assess the possibility of fitting all animals into GWAS at once, and avoid division into smaller groups, we tested two software designed for handling large-scale data. The software were tested on *Bos taurus* autosome (BTA) 14 and for the trait MY. The fastGWA application [20], implemented in GCTA [39] is a resource-efficient, mixed linear model (MLM) based tool, which utilizes a sparse GRM to account for relatedness [20]. The sparse GRM was created from autosomal 50K SNP chip full-dense GRM with --make-bK-sparse 0.05 that sets all off-diagonal elements less than 0.05 to 0. GWAS was run on 679,933 markers on BTA14 and 180,217 individuals, with MAF filtering for variants lower than 0.01, using the sparse GRM and --fastGWA-mlm command.

SAIGE, an R-based scalable and accurate generalized mixed model tool [21] that efficiently performs on both binary and quantitative traits, is able to handle large datasets, and can account for sample relatedness. The generalized mixed linear model used here can be described as follows:

2 $${\mathbf{g}}\left( {{\mathbf{y}}_{{\mathbf{i}}} } \right) = { }{\mathbf{X}}_{{\mathbf{i}}} {{\varvec{\upalpha}}} + { }{\mathbf{b}}_{{\mathbf{i}}} + { }{{\varvec{\upvarepsilon}}}_{{\mathbf{i}}} {\mathbf{,}}$$

where ${\mathbf{y}}_{\mathbf{i}}$ is a vector of phenotypes for the *ith* individual; 1 × (1 + *p*) ${\mathbf{X}}_{\mathbf{i}}$ represents *p* covariates including the intercept; ${\varvec{\upalpha}}$ is a (1 + *p*) × 1 vector of fixed effects; ${\mathbf{b}}_{\mathbf{i}}$ is a random effects vector with distribution $\text{N }(0, \tau \psi )$, where N denotes sample size, $\psi$ is an N × N GRM, and $\tau$ is the additive variance, and finally, ${{\varvec{\upvarepsilon}}}_{\mathbf{i}}$ is a vector of random residual errors [21]. We used SAIGE v. 1.3.1 and R v. 4.3.3 to perform the analyses. The first step included fitting of a null linear mixed model using a full GRM calculated from a 50K SNP chip. The first four principal components (PC) from BTA14 were extracted using the approximation method [46] implemented in PLINK v. 2.0 [47], as recommended for large samples, and included as covariates. Before calculating the PCs, variants in high linkage disequilibrium on BTA14 were pruned with PLINK v. 1.9 [48], based on pairwise *R*^2^ correlation greater than 0.1 (--indep-pairwise 50 10 0.1). We performed a single-variant test on BTA14 with LOCO = FALSE, and default quality control settings including MAF = 0 and minor allele count (MAC) of 20. Additionally, we tested the performance of the method with filtering for MAF = 0.01 and MAC = 3605, to eliminate rare variants. Analyses were performed using the scripts provided by the software developers; “step1_fitNULLGLMM.R” and “step2_SPAtests.R”. More details are available at https://saigegit.github.io/SAIGE-doc/docs/single.html.

### Downstream analyses

To identify potential causal variants among the genome-wide significantly associated variants, fine-mapping of independent QTL regions, and additionally, of all significant signals per chromosome, was conducted with BFMAP v. 0.65 [49]. Independent regions for fine-mapping were determined with PLINK v. 1.9 [48] clumping analysis on genome-wide significant variants. The parameters applied included an LD threshold of 0.2 and a physical distance threshold for clumping of 500 kb. Fine-mapping was carried out for all 180,217 samples. BFMAP is a Bayesian-based software tool that utilizes a forward selection approach, including adding independent signals in the model, repositioning signals, and generating a credible list of variants for each association signal [49]. Each variant in the credible set is also assigned with a posterior probability of causality (PPC).

SnpEff [50] and SnpSift [51] were utilized for the functional annotation of credible variant sets and prediction of their effect on genes, as well as the identification of the closest genes. Candidate regions were investigated through the Animal Quantitative Trait Loci database (Animal QTLdb) Release 54 (last accessed 2 November 2024), which reports the known candidate variants and genes [52], and using publications previously associated with milk production traits. A BLAST/BLAT [53, 54] search from Ensembl release 112 was used to make a comparison of transcript sequences against the human genome. Venn diagrams of common candidate variants were created using the R package VennDiagram [55]. Functional-And-Evolutionary Trait Heritability (FAETH) scores [24] were assigned to potential causal variants. Xiang and colleagues [24] established the FAETH framework by performing multiomics analyses of large cattle datasets. Ruidong Xiang provided us with FAETH scores and variant categories. Xiang et al. [24] estimated the variance explained by 13 variant categories across 34 complex traits in dairy cattle, and calculated the FAETH scores of more than 17 million sequence variants based on their expected contribution to genetic variance, by combining the results from all traits and all variant categories. Variant categories included both experimental and previously published datasets. Categories from [24] used for annotation and ranking of variants in this paper included: exon expression QTLs (eeQTLs), gene expression QTLs (geQTLs), and splicing QTLs (sQTLs) discovered from the liver, muscle, white blood, and milk cells as published in [56], allele-specific expression QTLs (aseQTLs) from white blood and milk cells [57], polar lipid metabolite QTLs (mQTLs) of various metabolite profiles from bovine milk fat [24], and chromatin immunoprecipitation sequencing (ChIPseq) data from liver [58], muscle [59] and mammary gland [24]. Xiang et al. [24] determined conserved sites (conserved) based on lifted over human genome sites and using the PhastCon software [60], according to information about conservation between 100 vertebrate species. The selection signature (selection.sig) category indicated variants with higher frequency in dairy than in beef breeds, detected from a multi-breed beef and dairy GWAS [24], and young variants (young) denoted variants that were the subject of recent selection, based on their proportion of positive correlations with rare variants [24]. Variants determined through fine-mapping that were present in Xiang’s dataset were given functional and evolutionary annotation and FAETH scores.

The percentage of genetic variance explained by the (1) credible sets generated by BFMAP, as well as by (2) top candidate variants, and by (3) random variants, was estimated using GCTA’s genomic-relatedness-based restricted maximum-likelihood (GREML) approach [61], by fitting the GRMs together in the model with 50K SNP chip variants. Random variants were chosen arbitrarily, across all autosomal chromosomes in a way that their number corresponded to the number of variants identified in all credible sets and top causal variants categories for each trait. The analysis was done for one of the four groups of ~45,055 animals due to high computational demand.

All the analyses were performed on the Scientific Compute Cluster (SCC), the high-performance computing system of the of the Gesellschaft für wissenschaftliche Datenverarbeitung mbH Göttingen (GWDG). The Scientific Linux 7.9 (Nitrogen) was used as an operating system with the x86_64 architecture and Intel(R) Xeon(R) Silver 4214 as a central processing unit (CPU) with a base frequency of 2.20 GHz. Performance of different GWAS software and BFMAP were assessed with Snakemake v. 7.22 [62] and with slurm built-in *sacct* command.

## Results

### Imputation

Imputation quality control was carried out by utilizing the DR2 parameter, built into the BEAGLE software. Markers imputed with DR2 < 0.75 were removed with BCFtools. Then, we checked the DR2 values of known causal variants, such as two variants in the *DGAT1* gene [63], which were imputed with almost perfect quality (DR2 = 0.99), as well as rs385640152 in the *GHR* gene [8] with DR2 = 0.98, and rs211210569 in *MGST1* [64, 65] with DR2 = 1.

### GWAS

A large number of variants exceeded the genome-wide significance threshold, regardless of the software used. Following are the results for each method that we used, with particular attention to genomic inflation and memory and time requirements utilized. First, we report the results of method performance testing (‘benchmarking’) on BTA14 and MY for each software, describing the computational requirements. Then we describe the detailed results of the method that showed the best performance, and in the end, we do a comparison of GWAS results obtained with different methods.

#### fastGWA

Using fastGWA, we were not able to obtain the results of GWAS on BTA14, due to both high memory and time requirements. After running for 120 h on a computing platform with 1.5 TB RAM (Random Access Memory) and 10 cores (Table 1), fastGWA did not manage to produce the results within the time limit of the HPC cluster. This process used a maximum resident set size (max RSS) of 723.176 GB and 4,321,150 s of CPU time (Table 1).

**Table 1:** Performance of different software and methods for GWAS of MY on BTA14

Software	Method	Sample number	Time (h:m:s)	Max RSS (GB)	CPU time (s)	Processor	CPU cores	Mem (GB)
GCTA	MLMA	45,055	23:01:40	85.148	820,581.06	2 × Xeon E5-2650 v4	12	512
GCTA	MLMA	180,217	120:00:28	1283.44	432,123	4 × Xeon E5-4620 v3	10	1536
GCTA	fastGWA (MLM)	180,217	120:00:19	723.176	4,321,150	4 × Xeon E5-4620 v3	10	1536
SAIGE	Null linear mixed model^a^	180,217	00:55:35	0.611	78,600	2 × Xeon Platinum 9242	48	384
SAIGE	Null linear mixed model^b^	180,217	00:53:34	0.605	77,136	2 × Xeon Platinum 9242	48	384

Method = GWAS method on which benchmarking was done (without GRM calculation step)

Sample number = number of samples utilized in analysis

Time (h:m:s) = wall clock time used to finish the analysis or to reach the set time limit (120 h)

Max RSS (GB) = max RSS in GB

CPU time (s) = CPU time in seconds

Processor = CPU & graphics processing unit (GPU) used for analysis

CPU cores = number of CPU cores per processor

Mem (GB) = memory in GB available per processor

^a^Default settings (MAF = 0, MAC = 20)

^b^Arbitrary settings (MAF = 0.01, MAC = 3605)

#### SAIGE

The GWAS for MY on BTA14 using SAIGE with default filtering settings identified 320,637 significant variants out of the 668,200 variants tested. We obtained GWAS results after running a generalized mixed linear model analysis for 55 min, on a 384 GB RAM platform with 48 cores (Table 1). The max RSS, denoting the peak amount of RAM the process held, was 0.611 GB (Table 1). A large number of variants had very low *p*-values, with top variant rs208417762, located within the *ADCK5* gene with a *p*-value of 1.3 × 10^–3467^. However, these very low *p*-values seemed to be the result of huge inflation (λ = 56.049). To check if different quality control parameters have an impact on inflation levels, we also run GWAS on BTA14 with MAF filtering of 0.01, and a MAC of 3605. This MAC cutoff was inferred based on previously obtained SAIGE’s summary statistics on BTA14 with default settings, where the variants with MAF of 0.01 had MAC of 3605. This resulted in a higher percentage of significant variants, with 307,853 significant signals out of 517,315 variants tested, and in even higher inflation (λ = 104.226) than when default settings were used. The top SNP was again rs208417762, with the same *p*-value of 1.3 × 10^–3467^. Computational resources were the same as for the default setting approach (Table 1).

#### GCTA and METAL

The MLMA approach in GCTA applied on 180,217 samples and BTA14 did not manage to produce results within 5 days on a 1536 GB RAM computing platform (Table 1).

Therefore, samples were divided into four random groups whose sizes ranged from 45,053 to 45,055, and MLMA was performed for each of the sample groups. For BTA14, this required 23 h of wall clock time and 820,581.06 s of CPU time on a computing platform with 512 GB and 12 CPU cores, as shown on the example of one of the groups in Table 1. The reason for dividing the samples into four groups was the fact that any division into smaller number of groups (e.g., two groups of ~90,000 samples or three groups of ~60,000 samples) failed to deliver the results within the 5-day time limit, similar as described when fitting all animals.

**Fig. 1:**
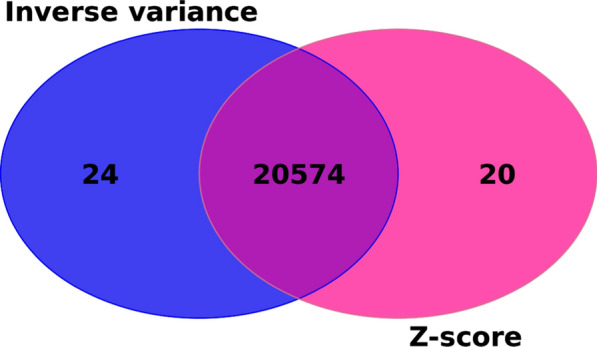
Concordant and discordant variants between inverse variance and z-score approach

This approach was subsequently applied to all autosomal chromosomes and all traits. Results obtained using GCTA’s MLMA on all autosomal chromosomes were merged using different METAL approaches and settings. Genome-wide significant variants and genomic inflation values of individual animal groups across all autosomes, before meta-analysis, are available in the Additional file 1: Table S3. The approaches used for meta-analysis were z-score and inverse variance, with and without sample size weighting and with fixed or random effects. Regardless of the meta-analysis approach used, the results were more or less the same regarding the number of significant variants and inflation levels, as shown with MY as an example (Table 2). Z-score and inverse variance approaches differed slightly in the number of significant variants, while all approaches gave the same level of genomic inflation (λ = 1.76). The variants that passed the genome-wide significance threshold were almost identical in both z-scores and inverse variance approach, despite the type of effect used and weighting. There was no difference in the number of significant variants within the z-score and the inverse-variance-based approach, regardless of additional settings applied (sample-size weighting, type of effects used). A small difference in the number of significant variants was observed when comparing z-score and inverse-variance-based approaches, both before and after genomic correction. Variants that remained significant after correction were nearly identical, with 20,574 variants in common between the two approaches (Fig. 1). Through the inverse-variance approach, we obtained 20,598 significant variants for MY, while z-scores gave 20,594 genome-wide significant markers. In total, 24 genome-wide significant variants were unique for the inverse-variance approach and 20 unique for the z-score approach. All of the approach-unique genome-wide significant variants appeared to be slightly below the Bonferroni threshold (2.897 × 10^–9^) in the other approach; for example, SNP rs210459588 had a *p*-value of 2.86 × 10^–9^ with inverse variance approach, and was significant there, while in the z-score approach the same SNP had a slightly higher *p*-value of 2.92 × 10^–9^ and was not significant there.

**Table 2:** Number of genome-wide significant variants and inflation factors obtained with different meta-analysis approaches for MY

Approach	λ, n_TOP_	λ_GC_, n_TOP_GC_
Z-score weighted, fixed effects	λ = 1.76, n_TOP_ = 54,032	λ_GC_ = 1, n_TOP_GC_ = 20,594
Z-score weighted, random effects	λ = 1.76, n_TOP_ = 54,032	λ_GC_ = 1, n_TOP_GC_ = 20,594
Z-score non-weighted, fixed effects	λ = 1.76, n_TOP_ = 54,032	λ_GC_ = 1, n_TOP_GC_ = 20,594
Z-score non-weighted, random effects	λ = 1.76, n_TOP_ = 54,032	λ_GC_ = 1, n_TOP_GC_ = 20,594
Inverse-variance weighted, fixed effects	λ = 1.76, n_TOP_ = 53,861	λ_GC_ = 1, n_TOP_GC_ = 20,598
Inverse-variance weighted, random effects	λ = 1.76, n_TOP_ = 53,861	λ_GC_ = 1, n_TOP_GC_ = 20,598
Inverse-variance non-weighted, fixed effects	λ = 1.76, n_TOP_ = 53,861	λ_GC_ = 1, n_TOP_GC_ = 20,598
Inverse-variance non-weighted, random effects	λ = 1.76, n_TOP_ = 53,861	λ_GC_ = 1, n_TOP_GC_ = 20,598

λ = genomic inflation factor

n_TOP_ = number of genome-wide significant variants

λ_GC_ = genomic inflation factor after genomic correction

n_TOP_GC_ = number of genome-wide significant variants after genomic correction

We proceeded with the weighted z-score approach with fixed effects across all autosomal chromosomes. Before applying correction for genomic inflation meta-analyzed GWAS datasets identified 54,032 significant variants for MY, 42,323 for FY, and 35,106 for PY, with the highest number of associations on chromosomes 5, 6, and 14. Low *p*-values were observed for many SNPs, with top variants positioned on the BTA14: rs109050667 (*p* = 7.04 × 10^–737^), rs136630297 (*p* = 7.18 × 10^–380^), and rs109050667 (*p* = 2.38 × 10^–221^) for MY, FY, and PY, respectively. Lambda values, calculated to assess for false associations were as follows: λ_MY_ = 1.76, λ_FY_ = 1.90, and λ_PY_ = 1.93. The reason for increased genomic inflation factors was due to the meta-analysis that inflated the *p*-values and therefore the number of genome-wide significant variants. To assess the effect of the meta-analysis on inflation we divided the individuals from direct-GWAS summary statistics into smaller groups, running the GWAS for each of these groups again, and merging them into a meta-analysis. The lambda values were higher after merging the animals into meta-analysis compared to direct GWAS summary statistics for the same individuals (see Additional file 2: Figure S1).

After applying post-meta-analysis genomic correction on all three traits, as implemented in METAL, 20,594 genome-wide significant variants remained for MY, 17,054 for FY, and 13,228 for PY. The top variants for all traits remained the same as before genomic correction, with somewhat higher *p*-values. The number of significant associations per chromosome, with *p*-values of top variants for each trait, are shown in Table 3.

**Table 3:** Number of significant variants per chromosome and top *p*-values for MY, FY, and PY

MY			FY			PY		
Chr	n_TOP_	*P*-value	Chr	n_TOP_	*P*-value	Chr	n_TOP_	*P*-value
3	375	1.371 × 10^–22^	2	3	2.098 × 10^–09^	5	882	1.044 × 10^–29^
5	4344	5.286 × 10^–79^	5	10,237	2.92 × 10^–104^	6	9571	1.7 × 10^–59^
6	4865	1.132 × 10^–59^	6	2775	8.877 × 10^–34^	11	277	9.977 × 10^–15^
10	113	2.85 × 10^–12^	14	3524	3.48 × 10^–201^	14	2421	8.65 × 10^–116^
11	469	1.345 × 10^–17^	15	28	4.049 × 10^–15^	19	30	5.053 × 10^–13^
14	6335	7.94 × 10^–419^	19	221	1.095 × 10^–17^	27	46	1.181 × 10^–11^
15	4	2.022 × 10^–09^	26	117	1.213 × 10^–13^	29	1	1.042 × 10^–09^
16	8	4.154 × 10^–10^	27	23	2.387 × 10^–12^			
19	21	4.967 × 10^–10^	28	126	1.079 × 10^–11^			
20	4041	1.898 × 10^–54^						
28	7	3.257 × 10^–10^						
29	12	6.657 × 10^–11^						

n_TOP_ = number of significant variants

**Fig. 2:**
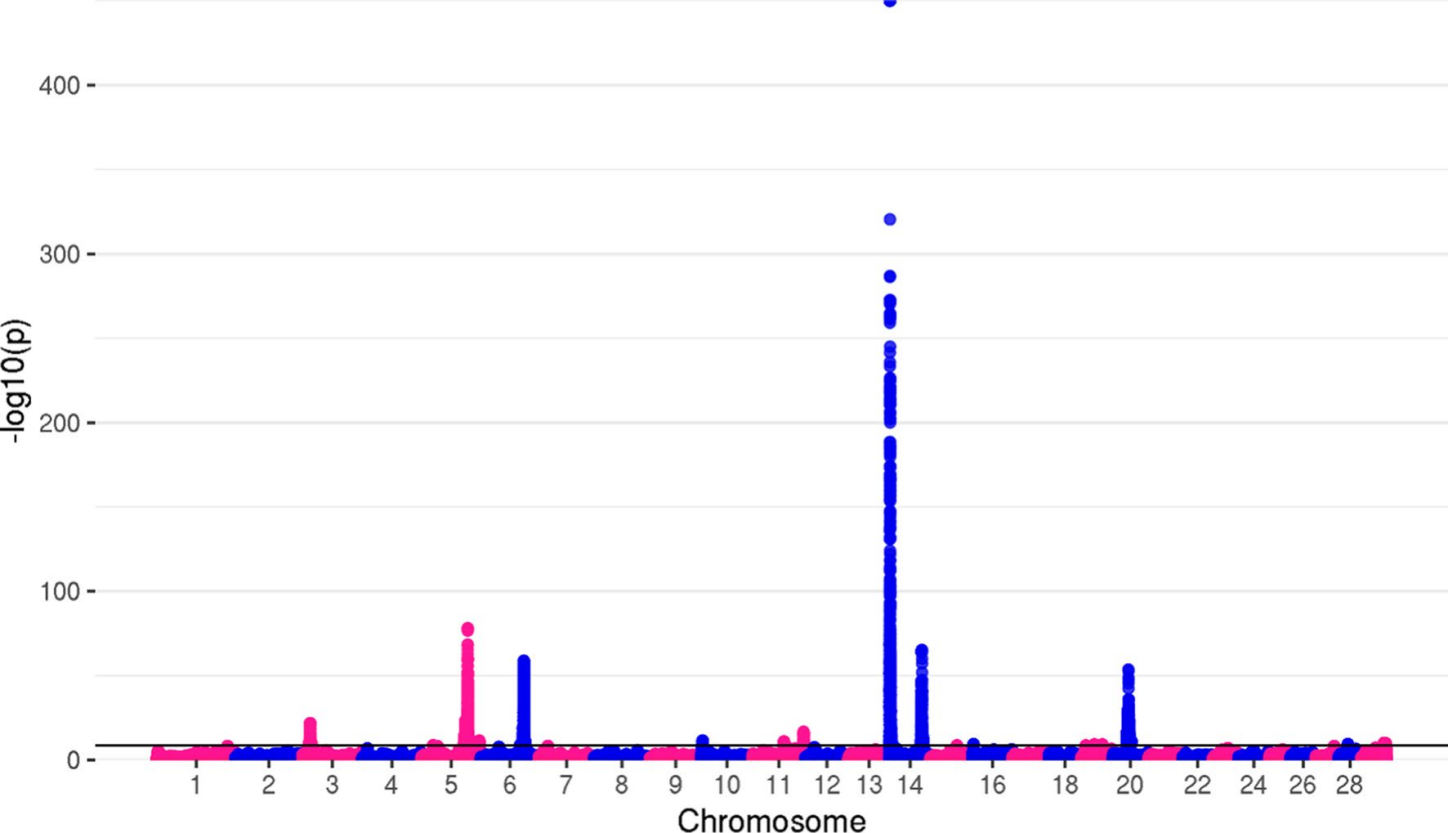
Manhattan plot for milk yield. The top genome-wide SNP (*p* = 7.94 × 10^–419^) for MY was located on BTA14. However, RStudio used for the creation of this plot was not able to show *p*-values < 3 × 10^–324^, reporting them as “0”

**Fig. 3:**
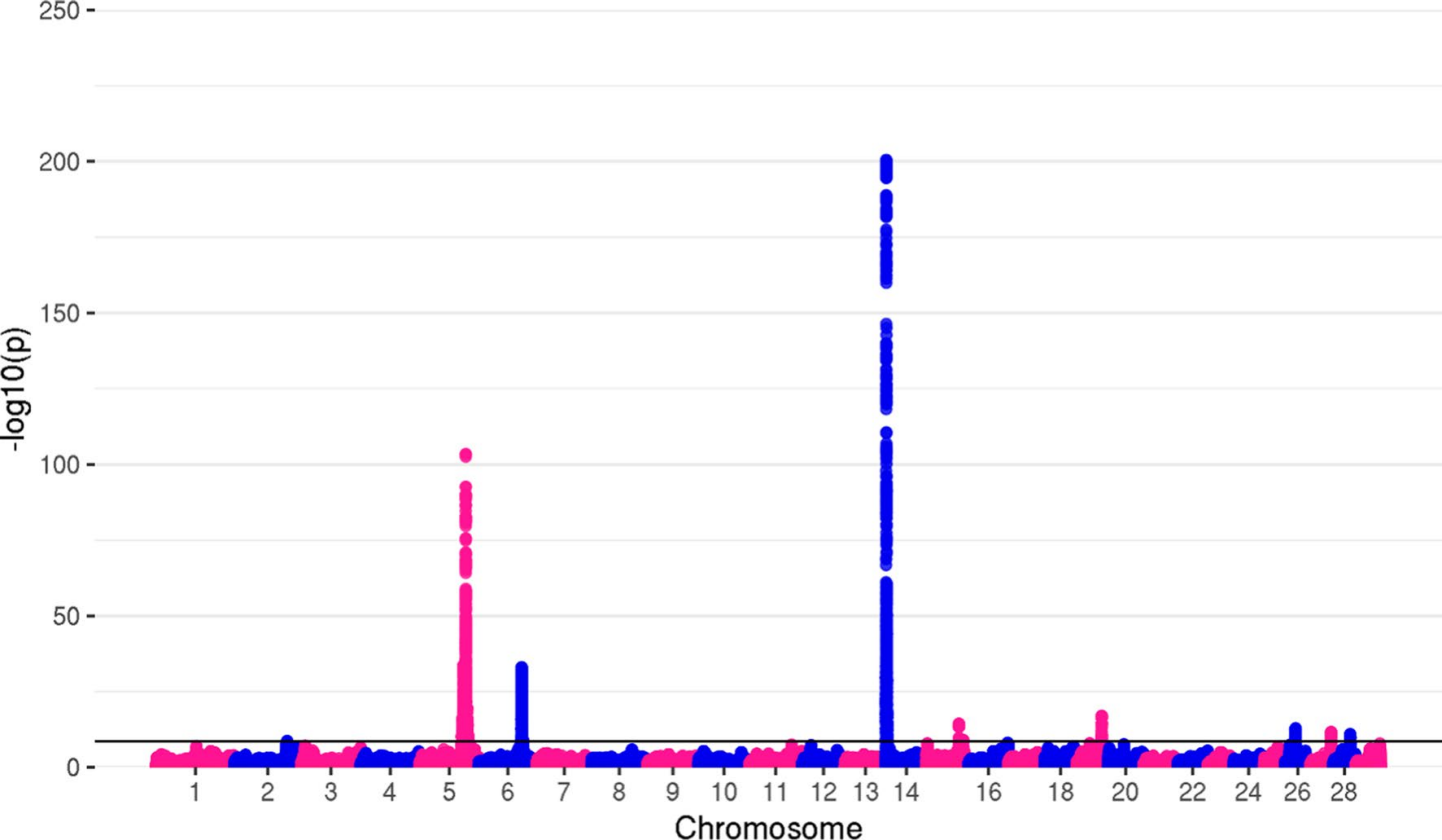
Manhattan plot for fat yield

**Fig. 4:**
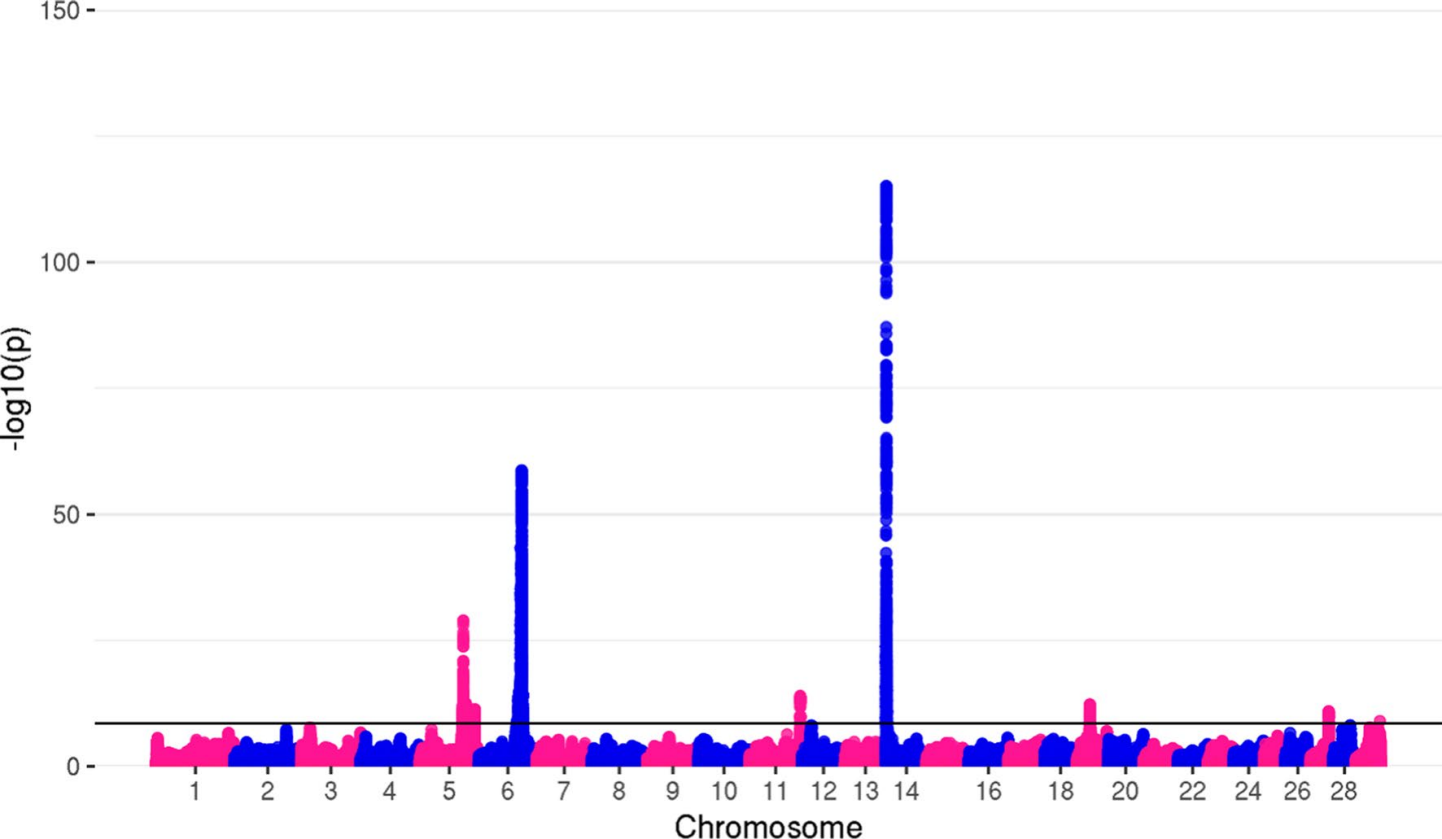
Manhattan plot for protein yield

Top variants were found in or in proximity to previously described milk production and composition genes. For MY, the top variants on chromosomes with the highest number of significant SNPs were located near or within *MGST1* [64–67] on BTA5, *GC* [68–70] and *NPFFR2* [71, 72] on BTA6, *ADCK5* [73–75], *CPSF1* [74, 76], *SLC52A2* [74], *SLC39A4* [74], *FBXL6* [75], *TMEM249* [75, 77] and *SCRT1* [78] on BTA14, and *GHR* [8, 79] on BTA20. For FY, top variants were located in or in the proximity of *MGST1* on BTA5, *GC* and *NPFFR2* on BTA6, and *CPSF1*, *SLC39A4*, *ADCK5*, *TMEM249*, *SCRT1*, *SLC52A2* and *FBXL6* on BTA14. The genes located within the most significant genomic regions for PY were: *ADCK5*, *CPSF1*, *FBXL6*, *SLC52A2*, *TMEM249* and *SLC39A4* on BTA14, *GC*, *NPFFR2*, *ENSBTAG00000049290* [80] and *SLC4A4* [72, 73] on BTA6, and *ABCC9* [72, 73, 81] on BTA5. Manhattan plots of GWAS results after genomic correction are shown in Figs. 2, 3 and 4.

**Fig. 5:**
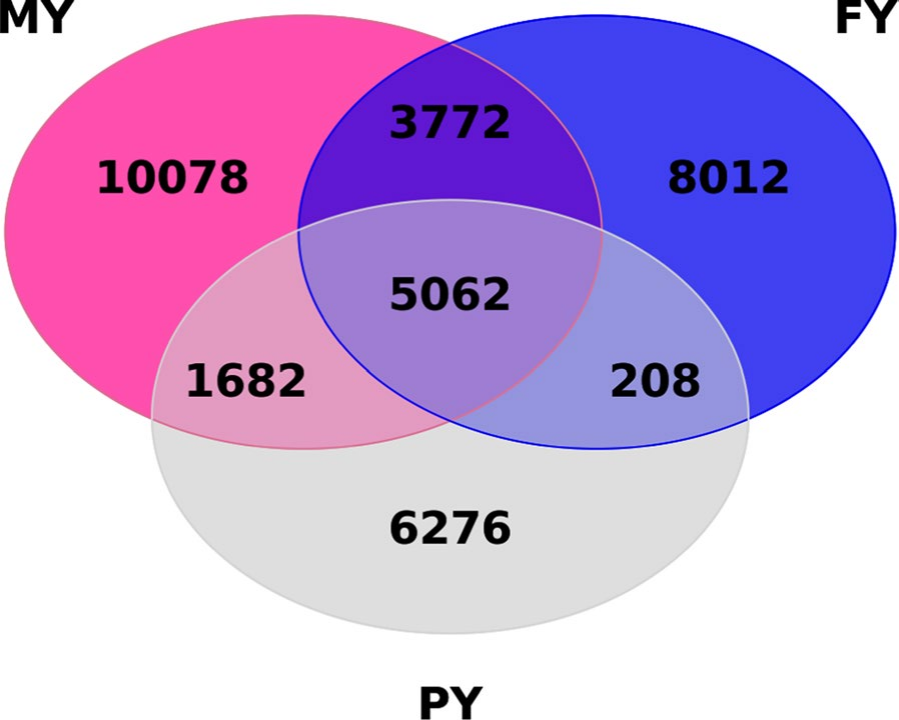
Venn diagram of MY, FY, and PY showing concordant and discordant genome-wide significant variants

Many variants were found to be associated with multiple traits, as shown on the Venn diagram (Fig. 5). The highest number of common candidate variants were found between MY and FY (8834). The second highest number of common candidate variants was between MY and PY (6744), 5270 variants were in common for FY and PY, and 5062 variants were in common for all three traits.

**Fig. 6:**
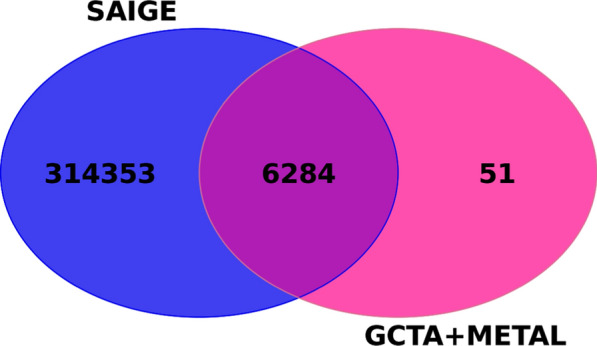
Venn diagram showing common genome-wide significant variants between SAIGE and GCTA + METAL approach

Additionally, as proof of concept, we examined if there is an overlap in significant variants on BTA14 between the GCTA and METAL-based approach and SAIGE. We describe results obtained on SAIGE with default quality control settings (MAF = 0, MAC = 20), since this approach resulted in smaller inflation, compared to the other one that filtered out rare variants. There were 6284 significant variants in common between GCTA combined with METAL and the SAIGE approach on BTA14 (Fig. 6), making almost all significant variants found with GCTA significant in the SAIGE approach as well. The Pearson correlation coefficient between the *p*-values of 6284 shared signals obtained with the two methods was 0.065, indicating a positive but very weak correlation.

### Downstream analyses

Genome-wide LD clumping resulted in a large number of independent regions (**≈**500 clumps across all the chromosomes and traits), whose fine-mapping was computationally unfeasible. Therefore, fine-mapping was applied to genome-wide significant variants, per chromosome. For example, fine-mapping of significant variants on BTA14 used more than 11 h of wall clock time, approximately 257 MB of peak memory usage, and 155,504 s of CPU time. BFMAP formed credible variant sets for each independent association, giving each variant PPC, which resulted in a list of more than 6000 candidate variants. The majority of variants were identified in introns or intergenic regions (see Additional file 1: Table S4). The number of predicted effects was larger than the actual number of variants, due to genes with multiple transcripts and variants which affect multiple genes. Regarding the variant impact on proteins, a high majority of variants were classified as modifiers, 33 variants had a moderate impact, and only one variant, found for PY (rs209618726), was high-impact. The list of all variants generated by BFMAP and their effects on proteins as well as closest genes, is available in Additional file 3: Table S5-S7. Variants with PPC ≥ 0.05 were further examined in more detail (see Additional file 4: Table S8). The majority of these variants were found on BTA5 (76), BTA14 (71) and BTA6 (48). These potential causal variants were located in or in proximity to 143 genes, of which the majority were known, while a few were previously undescribed genes for milk production traits (see Additional file 4: Table S8). Some of the known genomic regions for milk production traits included *MGST1*, *SLC15A5* and *ABCC9* on BTA5, *GC* and *NPFFR2* on BTA6, *ADCK5* and *CPSF1* on BTA14, *STAT5B* on BTA19, and *GHR* with a known causal variant for milk yield and composition, rs385640152 [8], ranked as the top causal variant with PPC ≈ 1 on BTA20 for MY. The fine-mapped associated candidate regions mainly corresponded to regions associated with GWAS top variants.

There were ~13 million variants in common between our imputed dataset and Xiang’s dataset, providing the functional annotation and FAETH score ranking to a reasonable variant number. Of 324 variants with PPC ≥ 0.05, we were able to assign FAETH scores to 205 variants (see Additional file 4: Table S8). The FAETH scores for these variants ranged from 3 to 17,341,551.5. Xiang et al. [24] considered all variants that were positioned within the top 1/3 of the FAETH score ranking as high ranked. Of 205 variants with assigned FAETH scores, 143 variants met these criteria. Of these, 98 variants fall into at least one of the functional and evolutionary variant sets, while 47 of them fall into more than one category. Variants with the highest FAETH score belonged to more than one functional and evolutionary category. Overall, the largest number of variants were assigned to sQTL (46) and ChIPseq (35) categories. 19 variants belonged to conserved sites, 26 to aseQTL, 20 to mQTL, 30 to eeQTL, 8 to geQTL, and 2 variants were categorized as young (see Additional file 4: Table S8). None of the 143 high-ranked variants was enriched in the selection.sig category. To our knowledge, of 143 variants with high FAETH scores, 65 were novel (Table 4) while others were previously reported for milk production traits in AnimalQTLdb.

**Table 4:** List of new candidate variants with highest FAETH ranking for MY, FY and PY

SNP ID	Chr	Pos	Trait	PPC	Gene ID	FAETH ranking	FAETH category
rs211682484	14	512,818	MY	1	*VPS28*	3712	eeQTL, mQTL, sQTL
rs211282001	19	26,084,321	PY	0.049	*DERL2*, *DERL2*-*DHX33*	5195	aseQTL, sQTL, conserved
rs482282570	14	515,265	FY	1	*VPS28*, *VPS28*-*ENSBTAG00000053637*	19,351.5	eeQTL, mQTL
rs43322204	2	107,437,210	FY	0.504	*ENSBTAG00000052917*	20,326	eeQTL, ChIPseq, conserved
rs109154013	2	107,436,884	FY	0.445	*ENSBTAG00000052917*, *GMPPA*, *GMPPA*-*ASIC4*	20,326	eeQTL, ChIPseq, conserved
rs110860915	14	269,611	FY	0.469	*ZNF16*	38,437	aseQTL, mQTL
rs42144935	28	34,583,251	FY	0.213	*ENSBTAG00000051468*-*ENSBTAG00000053285*	73,845.5	conserved
rs379835038	16	1,790,685	MY	0.164	*ENSBTAG00000052913*-*SOX13*	103,244	sQTL, conserved
rs42364317	15	73,957,297	FY	0.157	*HSD17B12*	115,542	conserved
rs379781983	19	42,251,886	MY	0.320	*RAB5C*, *DHX58*, *KAT2A*	121,117.5	conserved
rs109914138	5	111,918,900	MY, PY	0.065, 0.126	*MRTFA*-*ENSBTAG00000042762*	130,714.5	conserved
rs110629954	6	86,624,871	FY	0.067	*SLC4A4*	170,586	conserved
rs383905919	11	63,499,172	MY	0.076	*RAB1A*	185,467	ChIPseq, conserved
rs132823555	14	301,588	MY	0.347	*ZNF16*-*C14H8orf33*	443,756.5	eeQTL, sQTL
rs133929619	6	85,437,733	MY	0.517	*CSN1S1*-*CSN2*	448,308	eeQTL, sQTL
rs110400525	6	85,437,683	MY	0.329	*CSN1S1*-*CSN2*	448,308	eeQTL,sQTL
rs43473266	6	86,442,746	FY	0.971	*SLC4A4*	479,810.5	eeQTL, sQTL
rs42193880	29	48,861,569	MY	0.083	*KCNQ1*	599,714.5	aseQTL, ChIPseq, sQTL
rs42193893	29	48,879,903	MY	0.185	*KCNQ1*	609,523	aseQTL, ChIPseq, sQTL
rs42193886	29	48,869,098	MY	0.593	*KCNQ1*	641,566.5	aseQTL, sQTL
rs208731717	29	48,867,561	MY	0.139	*KCNQ1*	641,566.5	aseQTL, sQTL
rs110195883	14	928,629	FY	0.143	*PLEC*-*EPPK1*	856,270	aseQTL, geQTL, ChIPseq
rs110611375	27	36,605,789	PY	0.268	*ENSBTAG00000054394*	894,797	sQTL
rs42129584	27	41,405,450	FY	0.046	*ENSBTAG00000024530*-*THRB*	902,737	eeQTL
rs109747060	27	41,403,593	FY	0.055	*ENSBTAG00000024530*-*THRB*	1,144,647.5	sQTL
rs207681942	27	41,404,656	FY	0.055	*ENSBTAG00000024530*-*THRB*	1,144,647.5	sQTL
rs110410005	6	85,445,513	PY	0.071	*CSN2, CSN1S1*-*CSN2*	1,297,266	aseQTL
rs42364319	15	73,957,731	FY	0.143	*HSD17B12*	1,323,213.5	eeQTL
rs137406385	11	103,249,124	PY	1	*ENSBTAG00000048091*-*PAEP*	1,837,811	sQTL
rs137024369	19	9,212,877	MY	0.5	*LPO*	1,850,755	young
rs41775103	15	65,312,559	FY	0.076	*EHF*-*APIP*	1,873,240.5	sQTL
rs109627258	6	86,569,048	FY	0.094	*SLC4A4*	1,906,212	sQTL
rs110579906	6	86,596,089	FY	0.074	*SLC4A4*	1,906,212	sQTL
rs109352307	6	86,606,598	FY	0.071	*SLC4A4*	1,906,212	sQTL
rs133025873	6	86,570,867	FY	0.068	*SLC4A4*	1,906,212	sQTL
rs43474193	6	86,611,800	FY	0.066	*SLC4A4*	1,906,212	sQTL
rs210484189	5	93,595,233	MY	0.045	*MGST1*-*SLC15A5*	1,985,008.5	eeQTL
rs380876919	15	53,307,805	MY	0.439	*MRPL48*	2,007,530.5	eeQTL
rs382481916	27	36,603,164	PY	0.308	*ENSBTAG00000054394*	2,297,071	
rs209058841	5	23,538,597	MY	0.147	*CRADD*	2,422,873.5	ChIPseq
rs109590923	6	87,080,314	MY	0.061	*GC*-*NPFFR2*	2,660,253.5	
rs110875064	6	85,438,156	PY	0.083	*CSN1S1*-*CSN2*	2,660,253.5	
rs110068670	6	85,438,122	PY	0.082	*CSN1S1*-*CSN2*	2,660,253.5	
rs110854433	6	85,439,641	PY	0.073	*CSN1S1*-*CSN2*	2,660,253.5	
rs208758483	5	23,540,335	MY	0.175	*CRADD*	3,380,312.5	
rs377917940	5	86,681,779	MY	0.056	*SOX5*-*ETNK1*	3,490,551.5	
rs134600906	6	87,020,005	PY	0.056	*GC*-*NPFFR2*	3,490,551.5	
rs110879981	6	87,028,643	PY	0.049	*GC*-*NPFFR2*	3,490,551.5	
rs42145023	28	34,605,826	FY	0.087	*ENSBTAG00000053285*-*ZMIZ1*	3,645,870	
rs208261425	5	23,521,846	MY	0.372	*CRADD*	3,757,434	
rs209881936	5	23,520,036	MY	0.155	*CRADD*	3,757,434	
rs210630350	5	23,521,422	MY	0.152	*CRADD*	3,757,434	
rs207809845	6	87,042,033	MY, PY	0.232, 0.172	*GC*-*NPFFR2*	3,941,230	
rs135062731	19	26,078,201	PY	0.235	*DERL2*	3,964,078	ChIPseq
rs385575388	27	41,388,251	FY	0.088	*ENSBTAG00000024530*-*THRB*	4,336,588	
rs209866818	28	18,583,595	MY	0.172	*ZNF365*-*ENSBTAG00000048611*	4,593,251.5	
rs41651420	28	18,585,274	MY	0.133	*ZNF365*-*ENSBTAG00000048611*	4,593,251.5	
rs207579654	28	18,588,395	MY	0.117	*ZNF365*-*ENSBTAG00000048611*	4,593,251.5	
rs210783863	28	18,588,283	MY	0.094	*ZNF365*-*ENSBTAG00000048611*	4,593,251.5	
rs108948567	11	63,518,954	MY	0.055	*RAB1A*-*ACTR2*	4,838,259	
rs109773024	11	63,518,909	MY	0.053	*RAB1A*-*ACTR2*	4,838,259	
rs381941220	11	63,518,918	MY	0.053	*RAB1A*-*ACTR2*	4,838,259	
rs208818003	28	18,597,601	MY	0.139	*ZNF365*-*ENSBTAG00000048611*	4,838,259	
rs41775116	15	65,278,877	FY	0.399	*EHF*-*APIP*	5,584,591.5	young
rs455107942	16	1,786,046	MY	0.064	*ENSBTAG00000052913*-*SOX13*	5,613,365	sQTL

The percentage of genetic variance explained by all credible sets variants, as well as by variants with PPC ≥ 0.05, was estimated for all three traits (Table 5). For MY, 4277 variants from 12 chromosomes explained 31.32% of the variance. Top candidate variants from 12 autosomal chromosomes explained 11.46% of the variance. For FY, 1035 credible sets variants from nine chromosomes accounted for 16.61% of the genetic variance, while the top 104 variants from seven chromosomes explained 7.74%. For PY, 10.29% of the variance was explained by 1122 of all variants generated by BFMAP from 7 chromosomes, and 3.93% was explained by 68 highest-ranking variants. Random variants explained 1.18% of the variance for 4277 arbitrarily chosen variants, 0.73% for 1035, 0.43% for 1122 variants, 0.13% for 178, 0.0001% for 104, and 0.14% for 68 variants.

**Table 5:** Genetic variance explained by top, random and all candidate variants for MY, FY, and PY

Trait	n_SNP_	V_TOP_	V_RANDOM_	SE_TOP_	SE_RANDOM_	n_SNP_	V_ALL_	V_RANDOM_	SE_ALL_	SE_RANDOM_
MY	178	0.115	0.001	0.018	0.001	4277	0.313	0.012	0.025	0.005
FY	104	0.077	0.000001	0.018	0.001	1035	0.166	0.007	0.024	0.003
PY	68	0.039	0.001	0.010	0.001	1122	0.103	0.004	0.021	0.002

n_SNP_ = number of variants incorporated into analysis

V_TOP_ = genetic variance explained by top causal variants (PPC ≥ 0.05)

V_RANDOM_ = genetic variance explained by random variants

SE_TOP_ = standard error of top causal variants

SE_RANDOM_ = standard error of random variants

V_ALL_ = genetic variance explained by all credible sets

SE_ALL_ = standard error of all credible sets

## Discussion

In this paper, we imputed and performed GWAS and fine-mapping using a huge amount of data, regarding both sample sizes and number of markers. Previously, Jiang et al. [72] and Liang et al. [82] analyzed an even larger number of cattle, however with a smaller amount of markers. Reynolds et al. [83] performed GWAS for milk traits on 124,000 cattle, being one of the first with similar sample size to ours. To our knowledge, this is the largest cattle GWAS to this day, taking into consideration both sample sizes and the number of markers analyzed. We present advantages and challenges encountered when working with this large amount of data.

### Imputation

We performed a stepwise imputation of 180,217 German Holstein cows from SNP chip up to sequence level. The stepwise imputation approach seems to improve the imputation accuracy, as previously shown in cattle [18, 84]. Imputation error rate tends to decrease when an intermediate reference panel is used [84], possibly due to a larger choice of possible haplotype matches between WGS and medium-density SNP chip, which are narrowed down when using an HD panel as an intermediate [18]. In our study, stepwise imputation was done using the Holstein breed HD panel, a subset from van den Berg et al. [31] as an intermediate step, and the WGS panel from the 1000 Bulls Genome Consortium, as a second step. The WGS-based panel consisted of various breeds of taurine cattle (see Additional file 1: Table S2). The usage of a multi-breed reference was shown to increase the imputation accuracy in many studies [85–88], especially for low-frequency variants [86]. However, multi-breed panels can be counter-productive if animals in the reference panel are too distant from the sample dataset [89, 90]. The usage of BEAGLE software for imputation can at least partly overcome this issue since its algorithms can prioritize between closer and genetically more distant individuals in the multi-breed reference panel [91]. Moreover, the 1000 Bulls reference panel consisted of a large number of Holstein animals (~1200) making them the most represented breed in the reference panel (see Additional file 1: Table S2), therefore enabling the reliable imputation of Holsteins even in the presence of genetically distant breeds. Another crucial factor to consider is the value used for the *N*_*e*_ parameter [91]. Default *N*_*e*_ in BEAGLE is 1,000,000, however, this corresponds to human populations for which it was initially developed. Therefore, updating the *N*_*e*_ parameter to smaller values such as we did here is needed, when working with other, less-diverse populations [91].

To evaluate the accuracy of imputation we used quality measure based on estimated genotypes (DR2) since SNP array genotyped animals were not whole genome sequenced. Stringent variant filtering based on DR2 is recommended [91]. Based on visual inspection of the variants’ DR2 values we decided to use a threshold of DR2 < 0.75. Known causal variants were retained in the dataset after DR2 filtering, and were imputed with near to perfect quality (DR2 = 0.98 to 1). Causal variants in *DGAT1* were among the 100 top genome-wide significant variants for all three traits analyzed but were not the top variants. A possible explanation for this could be the presence of additional variation in the form of a known variable number of tandem repeats (VNTR) in the *DGAT1* region [87, 92].

### GWAS

Growing number of animals are being routinely genotyped for genomic prediction, providing an opportunity for subsequent imputation and GWAS. However, the growing sample sizes and marker amounts are not fully met by advances in software development, making it challenging to perform GWAS on large-scale data. Mixed linear model-based methods often suffer from extensive computational running times that, depending on the algorithm used, are heavily affected by both sample and marker numbers [20, 93]. Recently, Jiang et al. [20] implemented the fastGWA tool in GCTA, to utilize large-scale data with reduced time complexity of approximately *O(MN)*, where *M* is the number of markers and *N* number of samples [20]. After running for a given time limit of 120 h, fastGWA’s MLM analysis did not manage to deliver the results on BTA14 on our dataset, probably due to a combination of a large number of both samples and markers. This process was extremely time and memory-demanding, occupying the full 1536 GB computing platform with exclusive access to its compute nodes (Table 1). At the moment when the analysis was canceled, the estimation of genetic variance had only just started. Since fastGWA application has been successful in human studies in even higher sample and marker sizes than ours [20], we believe that higher relatedness in cattle datasets could resulted in longer variance estimation runtime. SAIGE, in contrast, does not use a GRM when performing a single-variant test, it rather uses a variance ratio computed in the first step of the analysis, resulting in a reduced computation time of *O(N)* [21]. Consequently, we obtained GWAS results after <1 h, on the 384 GB computing platform (Table 1). Unfortunately, *p*-values were severely inflated, therefore we took the results with great caution. When run with default settings, inflation although high (λ = 56.049), was half-size smaller then when filtering out the rare variants (λ = 104.226).The potential cause of inflation might be the type of phenotype measurements that we used, but also the different structure of non-human samples, in our case. Previous GWAS analyses [94–96] performed using SAIGE were carried out for mainly binary traits on human populations, therefore making direct comparison unfeasible. The best approach seems to be GCTA’s MLMA which utilizes the whole GRM constructed from the 50K SNP chip. MLMA uses *O(MN*^2^*)* for association statistics calculation, resulting in extended runtime [20, 97]. As a result, we were not able to run GWAS on all animals at once, therefore individual GWAS summary statistics on smaller groups were pooled into a meta-analysis. Due to the large sample sizes in our study, which might contribute to the rise in genomic inflation [98], lambda values were measured before (see Additional file 1: Table S3) and after performing the meta-analysis. Genomic inflation denotes spurious associations between variants and a trait, where the relationship between a phenotype and a SNP seems to arise from different factors than the true association [99]. These factors include population stratification [100], cryptic relatedness [101], polygenic inheritance [99], or strong association between variant and phenotype [102]. Although some of the genomic inflation in our study might be attributed to the polygenicity of milk production traits [103], and population structure in German Holstein [104], the main source of genomic inflation was the use of meta-analysis software (see Additional file 2: Figure S1). Similar findings were reported in human studies [105], where a large number of individuals are often pooled into the meta-analysis. The use of meta-analysis was inevitable in our case, due to the large samples that our HPC cluster was not able to utilize. MLMA accounted properly for genomic inflation, as the direct GWAS summary statistic had lambda values ranging from 0.962 to 1.026 (see Additional file 1: Table S3), and values up to 1 are usually considered as acceptable for genomic inflation. To prove that inflation was not due to population structure amplification that might arise when pooling the samples into the meta-analysis [106], we divided one of the animal groups on which we obtained summary statistics. After the animals were divided into two groups, GWAS was run for each of them again. Then, after obtaining the summary statistics, two groups of samples are merged into the meta-analysis. As shown in Additional file 2: Figure S1, lambda values for the same samples were increased after combining them in a meta-analysis. Moreover, an increase in the number of animal groups combined in a meta-analysis led to higher genomic inflation.

We evaluated two meta-analysis approaches implemented in METAL, with special focus on genomic inflation values. The z-score approach utilizes the *p*-value and direction of effect to calculate z-scores, while the inverse-variance approach weights beta coefficients using their estimated standard errors and gives weighted effect size estimates as an output [23]. The z-score and inverse variance approach gave identical values of genomic inflation (λ = 1.76), no matter the type of effect used and whether the sample size weighting was used or not (Table 2). A small difference in the number of significant variants was observed when comparing the two approaches (Fig. 1). It has been shown before that the z-score and inverse variance approaches give similar results [107]. Previous meta-analyses on cattle performed with METAL used predominantly the z-score approach [108–110]. Even though inverse-variance gave almost identical results to z-scores in our study, the former is considered the proper one when combining independent effects [111]. Therefore, we opted for the z-score approach, given that our samples came from the same initial dataset which we split solely for the purpose of performing the GWAS. Within the two basic approaches, there was no difference when using fixed and random effects, indicating the absence of heterogeneity between the groups combined [112]. The fixed effects model assumes one true effect underlying all the studies, i.e., the same effect of variant across all studies, while the random effects model assumes that the true effect varies [113, 114]. The fixed effect method was therefore the correct approach to proceed with, given the similar effect of variants between groups [115]. Even though there was no difference with and without sample-size weighting in our case (Table 2), given the equal sample sizes across the groups, sample-size weighting in the z-score approach is shown to be the preferable meta-analysis method [116], especially when allele frequencies between groups do not differ [107].

### Downstream analyses

Fine-mapping is the usual next step after obtaining summary statistics from GWAS. The top variants identified with GWAS are not always necessarily the true causal variants, but rather in LD with causal variants. To infer potential causal variants among genome-wide significant variants, different fine-mapping methods have been developed (reviewed by [117]). BFMAP employs the Bayesian approach for fine-mapping and has previously demonstrated good performance in cattle [118, 119]. Identification of independent QTL regions through conditional analyses such as the GCTA’s COJO [120] or PLINK’s clumping [48] is usually done before carrying out fine-mapping since it is computationally more efficient to perform fine-mapping at one region at a time [117]. Due to a large number of independent QTL regions, and high memory requirements of the fine-mapping procedure, in our case, it was not possible to fine-map all independent regions. Instead, we employed fine-mapping of all genome-wide significant variants per chromosome. This way we were able to obtain a list of potential causal variants, even though we probably missed some due to the large number of variants the fine-mapping software had to inspect at once. However, BFMAP uses a forward selection approach, which determines independent association signals within candidate regions and forms credible sets for each independent signal [49], therefore, we believe, partially circumventing our inability to fine-map each independent region separately. After carrying out the fine-mapping, candidate variants and genes were retrieved by searching public databases such as Animal QTLdb and reviewing journal papers on previously reported candidate genes and QTLs. We confirmed many of the previously reported candidate variants and candidate genes for milk production and composition (see Additional file 3 and Additional file 4), but also discovered new, previously unreported loci (Table 4). For simplification, we discuss only candidate genes associated with the variants with PPC ≥ 0.05, while the list of all associations can be found in Additional file 3: Tables S5–S7. The majority of the variants found in all credible sets were intronic and intergenic (see Additional file 1: Table S4). Most of the variants were, therefore, non-coding, which is in line with the majority of other GWAS publications [66, 121, 122]. Nayeri et al. [66] showed that a large proportion of the most significant variants affecting milk yield and composition traits in Holstein and Jersey cattle were located in non-coding regions of the genome. Both intron and intergenic variants usually do not code for proteins, making their functional prediction challenging [123]. However, recent research in human studies (reviewed by [121]) and cattle [124] has shown that even the variants in non-coding regions may play an important part in complex traits and diseases, by indirect involvement in gene expression regulation. Known QTNs in livestock are not all coding variants that cause a change in amino acid [6, 125], therefore, variants in non-coding regions can be causal as well [124]. Xiang et al. [24] showed that non-coding variants can contribute substantially to variance in complex traits in cattle. After the identification of candidate variants through fine-mapping, experimental validation is required for variants to be considered causal. For this purpose, prioritization of genome-wide significant variants according to external evolutionary and functional information [24] is suggested as the next step, followed by sequencing and gene editing experiments. In order to prioritize the potential causal variants and predict their possible effect on phenotype, variants with PPC ≥ 0.05 were functionally annotated and assigned FAETH scores. Xiang et al. [24] estimated the variance explained by 13 variant categories across 34 complex traits in dairy cattle, and calculated the FAETH score for more than 17 million sequence variants based on their expected contribution to genetic variance, by combining the results from all traits and all variant categories. This way one can rank the variants and infer their potential effect on the trait. Variant categories with the highest heritability estimates were conserved sites and mQTLs, followed by eeQTLs, sQTLs, geQTLs, and aseQTLs [24]. In our case, variants with a PPC lower than 0.1, were categorized as the variants with the highest FAETH scores, making the PPC ≥ 0.05 a reasonable cutoff. The variants with top FAETH scores were some of the known, previously described candidate variants for milk production and/or composition such as rs41256919 [126] and rs135473276 [73] at BTA14 within the *MAF1* gene, rs110126359 [127] in *GPAA1* and rs137070163 [127] within *CYHR1* on BTA14, and others. Among the variants with PPC ≥ 0.05, we did further filtering to include only variants with high FAETH scores (up to 5.9 million ranking). There were 65 new, previously unreported SNPs with PPC ≥ 0.05 and high FAETH scores (Table 4). Several variants without rsIDs were found among the top candidates, however, we do not report these in the main text, since it was hard to infer whether they were reported previously or not. For FY, we identified 22 variants, for MY 32, and 13 for PY. Two variants were in common for MY and PY. All of the 65 novel candidate causal variants were non-coding, making the conclusion about their biological consequences hard. However, they also had high FAETH scores (Table 4), and were enriched in many functional (eeQTL, mQTL, sQTL, aseQTL, ChIPseq) and evolutionary (conserved) categories. Expression QTLs (eQTLs) represent variants associated with gene expression levels [128]. aseQTLs quantify differences in expression between the two parental alleles at heterozygous sites [129], sQTLs affect alternative splicing [130, 131] while mQTLs denote variants affecting the levels of metabolites [132]. Membership in these categories suggests that variants and the genes found in their proximity have greater potential to be functionally associated with traits of interest. Similar relationships have been shown previously in cattle [133–136]. The majority of the genes have been previously reported for milk production traits in cattle (Table 4), so our results do confirm previous findings in a huge data set. Given the functional and evolutionary evidence found for particular variants related to these genes, our results furthermore contribute to the understanding of how those genes might be involved in trait expression.

However, there were also variants found within six novel genes (*ENSBTAG00000024530*, *ENSBTAG00000048611*, *ENSBTAG00000051468*, *ENSBTAG00000052913*, *ENSBTAG00000052917*, and *ENSBTAG00000053285*). Three genes (*ENSBTAG00000048611*, *ENSBTAG00000051468*, and *ENSBTAG00000053285*) are long non-coding RNA (lncRNA) genes, which were present in the Ensembl database up to release 110, but have now been removed. To find further information about the remaining three genes, we subjected the respective transcript sequences to an Ensembl’s BLAST/BLAT search against the human genome (Table 6).

**Table 6:** BLAST/BLAT search results for three genes associated with top causal variants

Chr & Pos	Gene ID	Gene type	Transcript	Most similar/overlapping human genes	Alignment score/E-value
27: 41,183,852–41,184,055	*ENSBTAG00000024530*	Processed pseudogene	*ENSBTAT00000012998.5*	*H2BC13*	329/3.0 × 10^–90^
16: 1,644,724–1,647,223	*ENSBTAG00000052913*	Protein- coding	*ENSBTAT00000085499.1*	*CACNA2D3, RPS15P5*	68/1.1 × 10^–11^
2: 107,434,43–107,435,891	*ENSBTAG00000052917*	Protein- coding	*ENSBTAT00000072763.1*	*GMPPA, ASIC4-AS1*	274/1.3 × 10^–73^

*ENSBTAG00000052913* is a protein coding gene, whose overlapping genes in human genome are *CACNA2D3* and the processed pseudogene *RPS15P5*. *CACNA2D3* (Calcium Voltage-Gated Channel Auxiliary Subunit Alpha2delta 3) was previously described as a candidate gene for protein yield in Holstein and Ayrshire [72, 137], yearling temperament in Angus [138], as well as for several reproductive and conformation traits including teat length and udder depth in Ayrshire cattle [137]. On BTA2 *ENSBTAG00000052917* overlapped with *ASIC4*-*AS1* and *GMPPA* (GDP-Mannose Pyrophosphorylase A), a gene that encodes *GMPPB* which catalyzes the synthesis of the nucleotide sugar GDP-mannose, required for glycosylation [139]. Gene expression of *GMPPA* was positively correlated with bovine milk fat globule size in the study of Huang et al. [140]. Taken altogether, these genes present interesting candidates for further research.

Except for the variants with high PPC and FAETH scores, it is worth mentioning one variant that was filtered out due to low PPC in previous steps. Stop-gain mutation rs209618726 (BTA6: 86,956,200 bp) in the *GC* gene was significant in PY GWAS, with a *p*-value of 1.6 × 10^–10^. The FAETH annotation was not available for this variant, however, given the previously described role of *GC* in milk production [68, 69], rs209618726 might be an interesting candidate for validation.

The percentage of trait variance explained by all credible set variants and top candidate variants or so-called SNP-based heritability [141] was calculated to see how much of the genetic variance is attributable to variants obtained through fine-mapping and to confirm the reliability of our fine-mapping procedure and findings. To account for common variants and avoid potential overestimation of variance, a GRM set up from 50K SNP chip data was included in the model. Both top and all credible set variants explained a large proportion of variance, especially when compared with random variants (Table 5), indicating the presence of causal variants among those and underpinning the infinitesimal model. There was a difference in the amount of variance explained between the top and all credible sets variants, with all credible set variants explaining twice as much variance. However, this is expected due to a larger number of variants present in all credible set categories. Variants associated with MY explained more variance than ones associated with FY and PY (Table 5), probably due to a larger number of variants incorporated into the analysis and higher heritability of MY.

By performing fine-mapping one can obtain the list of potential causal variants, and this is usually followed by validation experiments, such as sequencing. In large GWAS such as ours, one cannot perform the sequencing of all candidate regions, since it is time-demanding and costly. Annotation of variants based on external sources can be useful here. We report new candidate variants, supported by external functional and evolutionary information based on Xiang et al. [24] and variance analyses.

## Conclusions

After performing large-scale GWAS and subsequent fine-mapping, we identified new candidate variants. Variants explained a comparatively large proportion of genetic variance and many ranked high when annotated with external functional and evolutionary information. In order to be able to fully exploit the power of GWAS, sequence data of very large samples are required, as shown in our study. Large samples can be both an advantage for obtaining new insights about the genetic architecture of complex traits, as well as a burden when it comes to handling and analyzing it efficiently. Our findings add to existing knowledge of milk production traits architecture and demonstrate the power of our data set and strategy. Future studies incorporating health traits and their relationship with milk production may leverage the power of this data to add to the improvement of animal welfare.

## Supplementary Information

Additional file 1: Table S1. Genotype arrays used for samples genotyping. Table S2. Composition of breeds of WGS reference panel. Table S3. Number of genome-wide significant variants and genomic inflation values of individual GWAS summary statistics. Table S4. All credible sets variant effects by type.

Additional file 2: Figure S1. Genomic inflation factors of MY measured on direct GWAS summary statistics before and after meta-analysis. To check the cause of genomic inflation in meta-analysis summary statistics, one of the animal groups on which we ran direct GWAS was divided into two groups. For each of the two groups, GWAS was run again, and summary statistics were merged into the meta-analysis. Lambda values obtained on meta-analysis summary statistics were higher (λ = 1.20) than ones measured for the same individuals on direct GWAS summary statistics (λ = 0.96). To further check the extent of inflation caused by meta-analysis, the same group of animals was divided again, this time, into four groups. GWAS was run for each of the groups and results were merged into the meta-analysis. Lambda values were even higher this time (λ = 1.57). The figure was created in BioRender. Falker-Gieske, C. (2025) https://BioRender.com/b52m739

Additional file 3. Table S5. Fine-mapping and functional annotation of all credible sets for MY. Table S6. Fine-mapping and functional annotation of all credible sets for FY. Table S7. Fine-mapping and functional annotation of all credible sets for PY.

Additional file 4. Table S8. List of variants with PPC ≥ 0.05 and their functional annotation.

## Data Availability

The SNP chip genotype data and deregressed proofs are not available because they are the property of the national computing center in Germany (Vereinigte Informationssysteme Tierhaltung w.V.). Imputed genotypes and summary statistics will be provided upon reasonable request.
