# Supplementary material for: Sequence-based GWAS in 180,000 German Holstein cattle reveals new candidate variants for milk production traits
Source: Genet Sel Evol. 2025 Feb 4;57:3. doi: 10.1186/s12711-025-00951-9 (PMC11796172; doi:10.1186/s12711-025-00951-9)
Supplement: Supplementary file 1 — Additional file 1: Table S1. Genotype arrays used for samples genotyping. Table S2. Composition of breeds of WGS reference panel. Table S3. Number of genome-wide significant variants and genomic inflation values of individual GWAS summary statistics. Table S4. All credible sets variant effects by type. [file 12711_2025_951_MOESM1_ESM.docx]

**Table S1 Genotype arrays used for samples genotyping**

| **Genotype array** | **SNP number on array** | **Number of animals genotyped** |
| --- | --- | --- |
| AffyVM2 | 63836 | 1 |
| AM3 | 61792 | 1 |
| AxiomMD V3 | 65004 | 487 |
| EuroG MD | 49331 | 4661 |
| EuroG MD V1.1 | 49852 | 2517 |
| EuroG MD V2 | 54271 | 1226 |
| EuroG MD V3 | 62902 | 3 |
| EuroG10K | 9072 | 440 |
| EuroG10K V1.1 | 9075 | 463 |
| EuroG10K V2 | 9001 | 614 |
| EuroG10K V4 | 11490 | 2856 |
| EuroG10K V5 | 13787 | 94423 |
| EuroG10K V7 | 13329 | 104594 |
| EuroG10K V8 | 13674 | 39156 |
| GGP3 | 26151 | 1 |
| GGP4 | 30105 | 27 |
| GMD | 47843 | 5 |
| Illumina 50k V2 | 54609 | 281 |
| Illumina 50k V3 | 53218 | 348 |
| Illumina LD | 6909 | 2 |
| WeatherbysVersa1.3 | 49629 | 95 |

**Table S2 Composition of breeds of WGS reference panel**

| **Breed** | **Animal number** |
| --- | --- |
| Abondance | 9 |
| Alentejana | 1 |
| Altai | 20 |
| ANAN | 2 |
| Angus | 401 |
| AngusGerman | 1 |
| AngusLowline | 4 |
| AngusRed | 33 |
| AngusSimmental | 1 |
| ARARCHAR | 1 |
| Aubrac | 9 |
| Auroch | 1 |
| AyrshireFinnish | 45 |
| BeefShorthorn | 8 |
| BelgianBlue | 9 |
| BelgianBlueHolstein | 4 |
| BelgianBlueLimousin | 1 |
| BelgianRedWhiteCampine | 10 |
| BelgiumBlue | 1 |
| BeltedCattle | 1 |
| BlondedAquitaine | 41 |
| Bohuskulla | 3 |
| Boskarin | 1 |
| Braunvieh | 6 |
| BrownSwiss | 294 |
| Buryat | 19 |
| Busa | 10 |
| Cabannina | 2 |
| Charolais | 154 |
| CharolaisAngus | 1 |
| CharolaisRedAngus | 1 |
| ChiAngus | 1 |
| Chianina | 15 |
| Cloned-polledDairyBull | 2 |
| Composite | 1 |
| Corriente | 4 |
| CostenoConCuernos | 2 |
| Crossbreed | 35 |
| Crossbreed(25%RedAngus;25%Simmental;25%Gelbvieh;25%Hereford) | 1 |
| Crossbreed(37.5%Gelbvieh;25%RedAngus;12.5%Simmental;12.5%Limousin) | 1 |
| Crossbreed(50%Simmental;25%RedAngus;25%Charolais) | 1 |
| Crossbreed(50%Simmental;37.5%RedAngus;12.5%Angus) | 1 |
| Crossbreed(50%Simmental;50%RedAngus) | 1 |
| Crossbreed(62.5%Angus;12.5%Simmental;12.5%Gelbvieh;12.5%Hereford) | 1 |
| Crossbreed(75%Gelbvieh;25%Limousin) | 1 |
| Crossbreed(HO62.5%;MO25%;JE12.5%) | 1 |
| DanishRedDairy | 4 |
| DanishRedDairyHolstein | 1 |
| DeepRedCattle | 9 |
| DeutschesSchwarzbuntesNiederungsrind | 56 |
| Devon | 1 |
| Dexter | 2 |
| DutchBelted | 11 |
| DutchFriesianRed | 11 |
| DutchImprovedRed | 9 |
| EasternBelgianRedWhite | 7 |
| EasternFinncattle | 15 |
| EasternFlandersWhiteRed | 12 |
| Eringer | 4 |
| Evolène | 3 |
| FinnishAyrshire | 12 |
| Fjäll | 11 |
| FjällCattle | 6 |
| Fleckvieh | 162 |
| FriesianJersey | 9 |
| Galloway | 1 |
| GallowayBelted | 3 |
| Gelbvieh | 52 |
| GermanRedAngler | 6 |
| GreyCattle | 6 |
| GroningenWhiteHeaded | 10 |
| Guernsey | 20 |
| Hanwoo | 22 |
| Hasake | 6 |
| HEAN | 5 |
| Hereford | 141 |
| HerefordMiniature | 2 |
| HerefordPolled | 4 |
| Hinterwaelder | 3 |
| Holstein | 1148 |
| HolsteinCharolais | 22 |
| HolsteinFriesian | 41 |
| HolsteinHereford | 9 |
| HolsteinLimousinF1Crossbred | 2 |
| HolsteinRed | 20 |
| HolsteinSimmental | 4 |
| HolsteinxJerseyF1Crossbred | 1 |
| IcelandicCattle | 5 |
| Illawarra | 1 |
| ImprovedRed | 1 |
| JapaneseNative | 8 |
| Jersey | 195 |
| JerseyHolstein | 42 |
| JerseyLimousin | 2 |
| Jutland | 5 |
| Kalmyk | 3 |
| Kalmykian | 10 |
| Kazakh | 9 |
| KazakhWhiteheaded | 5 |
| Kholmogory | 32 |
| LatvianBrown | 10 |
| Limia | 1 |
| Limonero | 6 |
| Limousin | 101 |
| LimousinBrownSwiss | 1 |
| LimousinHereford | 2 |
| LimousinHolstein | 1 |
| LimousinSimmental | 2 |
| LithuanianRed | 12 |
| Lowline | 1 |
| Luxi | 1 |
| MAARCTwinner | 4 |
| MaineAnjou | 22 |
| Marchigiana | 9 |
| Maremmana | 1 |
| Maronesa | 1 |
| Menggu | 11 |
| MeuseRhineYssel | 25 |
| ModernAngler | 20 |
| ModernDanishRed | 54 |
| Mongolian | 4 |
| Montbeliarde | 63 |
| MurrayGrey | 2 |
| MWFDEU | 3 |
| Normande | 44 |
| NorthernFinncattle | 19 |
| NorwegianRed | 347 |
| OriginalBraunvieh | 125 |
| Ottonese | 2 |
| Pajuna | 1 |
| Parthenaise | 2 |
| PezzataRossaItaliana | 1 |
| Piedmontese | 10 |
| PiedmonteseNormande | 1 |
| Pinzgauer | 1 |
| PodolianSerbia | 10 |
| Podolica | 1 |
| PolishRed | 7 |
| RedAngus | 2 |
| RedDairy | 2 |
| RedWhiteDualPurpose | 17 |
| Rendena | 2 |
| Ringamålako | 8 |
| Rödkulla | 9 |
| Romagnola | 25 |
| RotesHöhenvieh | 6 |
| RougeDesPres | 9 |
| Salers | 25 |
| SanMartinero | 2 |
| Sayaguesa | 1 |
| ScottishHighland | 7 |
| Shorthorn | 33 |
| Sikias | 1 |
| Simmental | 137 |
| SimmentalAngus | 1 |
| SimmentalFleckviehPezzatarossa | 36 |
| SMSMCHAR | 1 |
| Stabilizer | 2 |
| SwedishPolled | 6 |
| SwedishRed | 50 |
| SwedishRedPolled | 6 |
| SwissFleckvieh | 10 |
| Tarentaise | 12 |
| TexasLonghorn | 3 |
| TraditionalAngler | 5 |
| TraditionalDanishRed | 15 |
| TraditionalLithuanianRed | 4 |
| TuranoMongolicus | 1 |
| Tuxer | 1 |
| TyroleanGrauvieh | 9 |
| TyroleanGrey | 17 |
| UkrainianGrey | 8 |
| Unknown | 199 |
| Väneko | 11 |
| Vorderwälder | 13 |
| Vosgienne | 4 |
| Wagyu | 29 |
| WagyuModern | 1 |
| WesternFinncattle | 15 |
| WestVlaamsRood | 11 |
| Xizang | 2 |
| Yakut | 44 |
| Yanbian | 11 |
| Yaroslavl | 22 |

**Table S3 Number of genome-wide significant variants and genomic inflation values of individual GWAS summary statistics**

|  | **1. group** | **2. group** | **3. group** | **4. group** |
| --- | --- | --- | --- | --- |
| **MY** | n_SNP_ = 7650, λ = 0.962 | n_SNP_ = 7751, λ = 0.970 | n_SNP_ = 7447, λ = 0.970 | n_SNP_ = 7828, λ = 0.963 |
| **FY** | n_SNP_ = 7507, λ = 0.999 | n_SNP_ = 6501, λ = 1 | n_SNP_ = 6053, λ = 0.998 | n_SNP_ = 6108, λ = 1.026 |
| **PY** | n_SNP_ = 4969, λ = 1.013 | n_SNP_ = 4866, λ = 1.020 | n_SNP_ = 3996, λ = 1.006 | n_SNP_ = 3314, λ = 1.021 |

n_SNP_ = number of genome-wide significant variants

**Table S4 All credible sets variant effects by type**

| **Predicted effect** | **MY** | **FY** | **PY** |
| --- | --- | --- | --- |
| 3 prime UTR variant | 18 | 24 | 22 |
| 5 prime UTR premature start codon gain variant | 5 | 0 | 0 |
| 5 prime UTR variant | 20 | 6 | 7 |
| Downstream gene variant | 230 | 94 | 208 |
| Intergenic region | 2845 | 643 | 532 |
| Intron variant | 1290 | 304 | 538 |
| Missense variant | 16 | 8 | 10 |
| Missense variant & splice region variant | 0 | 1 | 0 |
| Non-coding transcript exon variant | 2 | 3 | 0 |
| Splice region variant | 1 | 0 | 0 |
| Splice region variant & intron variant | 2 | 0 | 3 |
| Stop gained | 0 | 0 | 1 |
| Synonymous variant | 20 | 15 | 16 |
| Upstream gene variant | 387 | 113 | 98 |
