# Supplementary material for: Sequence-based GWAS in 180,000 German Holstein cattle reveals new candidate variants for milk production traits
Source: Genet Sel Evol. 2025 Feb 4;57:3. doi: 10.1186/s12711-025-00951-9 (PMC11796172; doi:10.1186/s12711-025-00951-9)

**Figure S1 Genomic inflation factors of MY measured on direct GWAS summary statistics before and after meta-analysis**


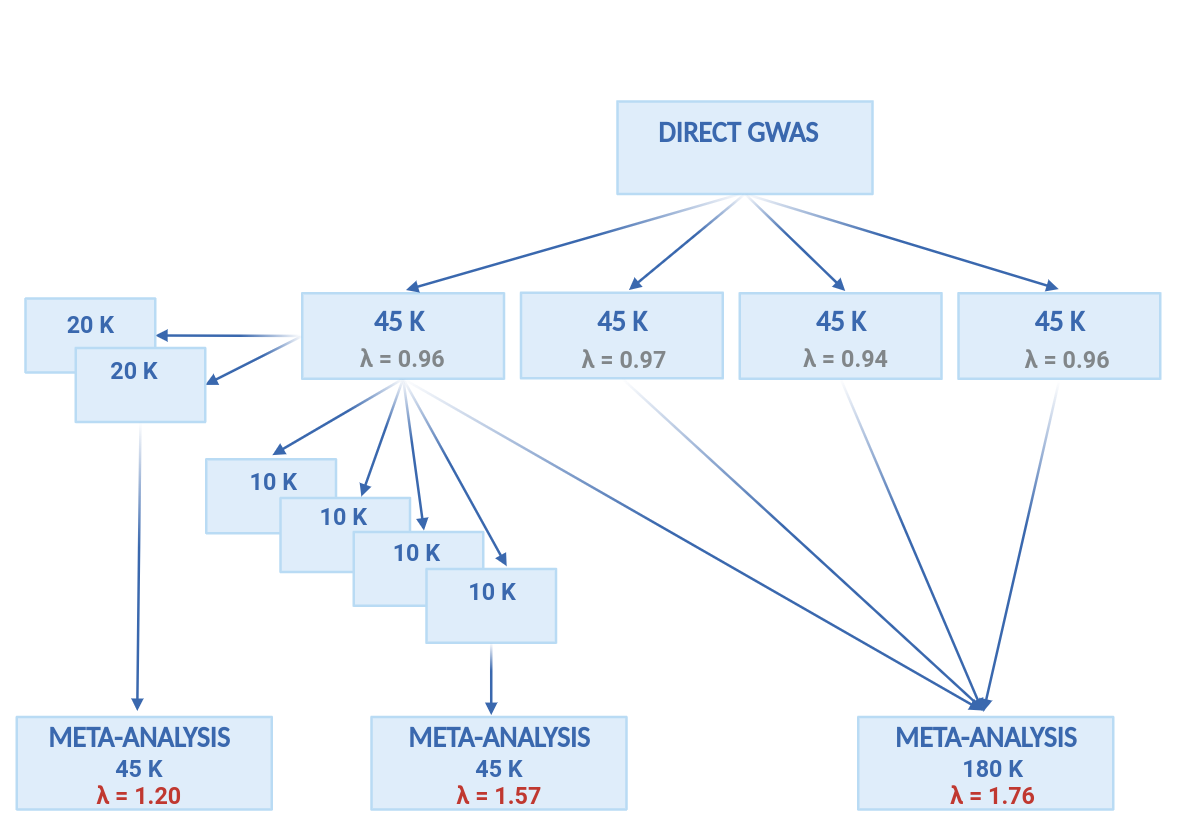

Supplement: Supplementary file 2 — Additional file 2: Figure S1. Genomic inflation factors of MY measured on direct GWAS summary statistics before and after meta-analysis. To check the cause of genomic inflation in meta-analysis summary statistics, one of the animal groups on which we ran direct GWAS was divided into two groups. For each of the two groups, GWAS was run again, and summary statistics were merged into the meta-analysis. Lambda values obtained on meta-analysis summary statistics were higher (λ = 1.20) than ones measured for the same individuals on direct GWAS summary statistics (λ = 0.96). To further check the extent of inflation caused by meta-analysis, the same group of animals was divided again, this time, into four groups. GWAS was run for each of the groups and results were merged into the meta-analysis. Lambda values were even higher this time (λ = 1.57). The figure was created in BioRender. Falker-Gieske, C. (2025) https://BioRender.com/b52m739 [file 12711_2025_951_MOESM2_ESM.docx]
